# Vascular inflammation and aortic stiffness: potential mechanisms of increased vascular risk in chronic obstructive pulmonary disease

**DOI:** 10.1186/s12931-018-0792-1

**Published:** 2018-05-24

**Authors:** Marie Fisk, Joseph Cheriyan, Divya Mohan, Carmel M. McEniery, Julia Forman, John R. Cockcroft, James H. F. Rudd, Ruth Tal-Singer, Nicholas S. Hopkinson, Michael I. Polkey, Ian B. Wilkinson

**Affiliations:** 1 Division of Experimental Medicine and Immunotherapeutics, University of Cambridge, Cambridge, UK; 2 Cambridge Clinical Trials Unit, Cambridge University Hospitals NHS Foundation Trust, Cambridge, UK; 3 NIHR Respiratory Biomedical Research Unit, Royal Brompton & Harefield NHS Foundation Trust and Imperial College, London, UK; 4 Department of Cardiology, Wales Heart Research Institute, Cardiff University, Cardiff, UK; 5 Division of Cardiovascular Medicine, University of Cambridge & Cambridge University Hospitals NHS Foundation Trust, Cambridge, UK; 6 GSK R&D, King of Prussia, Pennsylvania, USA

**Keywords:** Chronic obstructive pulmonary disease, α1 antitrypsin deficiency, Vascular inflammation, Aortic stiffness, Positron emission tomography

## Abstract

**Background:**

Chronic obstructive pulmonary disease (COPD) is a complex inflammatory condition in which an important extra-pulmonary manifestation is cardiovascular disease. We hypothesized that COPD patients would have increased aortic inflammation and stiffness, as candidate mechanisms mediating increased cardiovascular risk, compared to two negative control groups: healthy never-smokers and smokers without COPD. We also studied patients with COPD due to alpha_− 1_ antitrypsin deficiency (α_1_ATD) as a comparator lung disease group.

**Methods:**

Participants underwent ^18^F-Fluorodeoxyglucose (FDG) positron emission tomography imaging to quantify aortic inflammation as the tissue-to-blood-ratio (TBR) of FDG uptake. Aortic stiffness was measured by carotid-femoral aortic pulse wave velocity (aPWV).

**Results:**

Eighty-five usual COPD (COPD due to smoking), 12 α_1_ATD-COPD patients and 12 each smokers and never-smokers were studied. There was no difference in pack years smoked between COPD patients and smokers (45 ± 25 vs 37 ± 19, *p* = 0.36), but α_1_ATD patients smoked significantly less (19 ± 11, *p* < 0.001 for both). By design, spirometry measures were lower in COPD and α_1_ATD-COPD patients compared to smokers and never-smokers. Aortic inflammation and stiffness were increased in COPD (TBR: 1.90 ± 0.38, aPWV: 9.9 ± 2.6 m/s) and α_1_ATD patients (TBR: 1.94 ± 0.43, aPWV: 9.5 ± 1.8 m/s) compared with smokers (TBR: 1.74 ± 0.30, aPWV: 7.8 ± 1.8 m/s, *p* < 0.05 all) and never-smokers (TBR: 1.71 ± 0.34, aPWV: 7.9 ± 1.7 m/s, *p* ≤ 0.05 all).

**Conclusions:**

In this cross-sectional prospective study, novel findings were that both usual COPD and α1ATD-COPD patients have increased aortic inflammation and stiffness compared to smoking and never-smoking controls, regardless of smoking history. These findings suggest that the presence of COPD lung disease per se may be associated with adverse aortic wall changes, and aortic inflammation and stiffening are potential mechanisms mediating increased vascular risk observed in COPD patients.

**Electronic supplementary material:**

The online version of this article (10.1186/s12931-018-0792-1) contains supplementary material, which is available to authorized users.

## Background

Chronic obstructive pulmonary disease (COPD) is a complex condition, associated with extra-pulmonary manifestations that contribute to poor health and mortality [1]. COPD patients have two-to-five fold increased cardiovascular risk compared to the general population, and cardiovascular disease (CVD) accounts for a third of all deaths in people with COPD [2, 3]. The precise mechanisms leading to increased cardiovascular risk in COPD patients are unclear. Smoking is an obvious shared risk factor, but several other mechanisms including systemic inflammation (which is the elevated levels of circulating inflammatory cells, cytokines or proteins, measured from the systemic circulation), potentially of pulmonary origin, and vascular inflammation and stiffening, may also be involved.

Inflammation plays an important role in the pathogenesis of both COPD and CVD, and increased markers of systemic inflammation, are associated with a poor prognosis in COPD [4, 5]. Local vascular inflammation measured by ^18^F-Fluorodexoyglucose positron emission tomography (FDG PET) imaging, is a promising vascular biomarker [6, 7] that correlates with atheromatous plaque macrophage content [8], and aortic inflammation quantified by FDG PET, has been shown to independently predict future cardiovascular events beyond the Framingham Risk Score (FRS) [9]. Interestingly, there are very few studies that have assessed vascular inflammation assessed by FDG PET in COPD patients. A small pilot study has previously reported increased aortic inflammation assessed by FDG PET in seven COPD patients compared to seven ex-smoker controls with normal spirometry. However, given that study groups were poorly matched for smoking exposure [10], it is uncertain whether observed differences were due to the presence of COPD per se or tobacco consumption.

Aortic stiffness defined by the gold standard of (carotid-femoral) aortic pulse wave velocity (aPWV) is also reported to be increased in COPD patients [11]. A recent meta-analysis indicates that aPWV predicts future cardiovascular events and mortality independently of traditional cardiovascular risk factors [12]. Increased aortic stiffness is itself associated with elevated systemic markers of inflammation in diverse conditions [13, 14], which may help explain why chronic inflammatory conditions such as rheumatoid arthritis have increased cardiovascular risk [15]. However, interestingly, we have recently shown in a large study of COPD patients, that despite having elevated systemic inflammatory markers, no independent relationship between aortic stiffness measured by aPWV and systemic inflammatory markers was observed [16]. Moreover, the relationship between anatomically localized vascular inflammation measured by FDG PET and both vascular stiffening and systemic inflammation in COPD patients, has not thus far been assessed.

We hypothesized that ‘usual COPD’ (COPD due to smoking) patients would have both increased aortic inflammation and aortic stiffness, independently of cigarette smoking. We also sought to determine the interaction of these vascular manifestations with lung disease per se, by also studying patients with COPD due to alpha-_1_ antitrypsin deficiency (α_1_ATD), as a positive lung disease control group. Furthermore, we examined the relationship between aortic inflammation measured by FDG PET with aortic stiffness and calcification, markers of systemic inflammation and measures of COPD severity, to better understand mechanisms of vascular risk in this complex condition.

## Methods

Data presented in this manuscript includes subjects recruited to the EVOLVE study (REC 13/EE/0165, UK CRN ID 1513) and EVOLUTION trial (NCT01541852). EVOLVE is a cross-sectional prospective FDG PET imaging study that was conducted in parallel to the EVOLUTION trial and included COPD patients with a plasma fibrinogen ≤2.8 g/l, α_1_ATD-COPD (Pi Z phenotype) patients, and smokers and never-smokers without COPD. EVOLUTION is a phase 2a randomized placebo-controlled trial of losmapimod in COPD patients with a plasma fibrinogen level > 2.8 g/L. Further information regarding the EVOLUTION trial has previously been published [17, 18]. In this manuscript, we have used the baseline data of these COPD patients recruited to the EVOLUTION trial in addition to COPD patients from EVOLVE to assess data from COPD patients with a range of plasma fibrinogen values, (plasma fibrinogen is a systemic inflammatory marker associated with increased cardiovascular risk [19, 20]), without incurring additional scans and associated ionizing radiation exposure. In addition, we have been able to longitudinally assess vascular FDG PET over a 4-month time period in COPD patients who received placebo as part of the trial.

Identical protocols were used for all subjects from both studies, for all data assessments presented in this manuscript, including scanning, hemodynamic measurements, and laboratory tests for example. In addition, the same equipment (e.g. scanners and machines), and study personnel conducted both studies. Both studies recruited subjects from two UK tertiary centers and received favorable opinions from the Cambridge South Research Ethics Committee. Written informed consent was obtained from all participants and the studies were carried out in accordance with institutional guidelines and the Declaration of Helsinki.

### Subject groups

There were four subject groups, which included 85 ‘usual’ COPD patients (with ≥10 pack years smoked history), 12 α_1_ATD-COPD patients (Pi Z phenotype), 12 smokers and 12 never-smokers, both with normal predicted spirometry. Smokers without COPD, had greater than a 10 pack year history, and smoked approximately > = 10 cigarettes per day in the preceding 12 months of study enrolment. Both COPD and α_1_ATD-COPD patients had to be clinically stable, and free of exacerbations in the preceding 4 weeks before enrolment in the study. Moreover, any other known chronic inflammatory conditions, a cardiovascular event in the preceding 6 months to study enrolment, and insulin dependent diabetes were the main exclusion criteria for all subject groups. The usual COPD patient group comprised baseline data (i.e prior to intervention of placebo or losmapimod) of 73 COPD patients with a plasma fibrinogen > 2.8 g/l enrolled in the EVOLUTION trial and 12 COPD patients with a fibrinogen ≤2.8 g/l. Age and gender were matched across the four groups as closely as possible to enable cross-sectional analysis of the different subject groups.

### Scan imaging and analysis

**Fig. 1:**
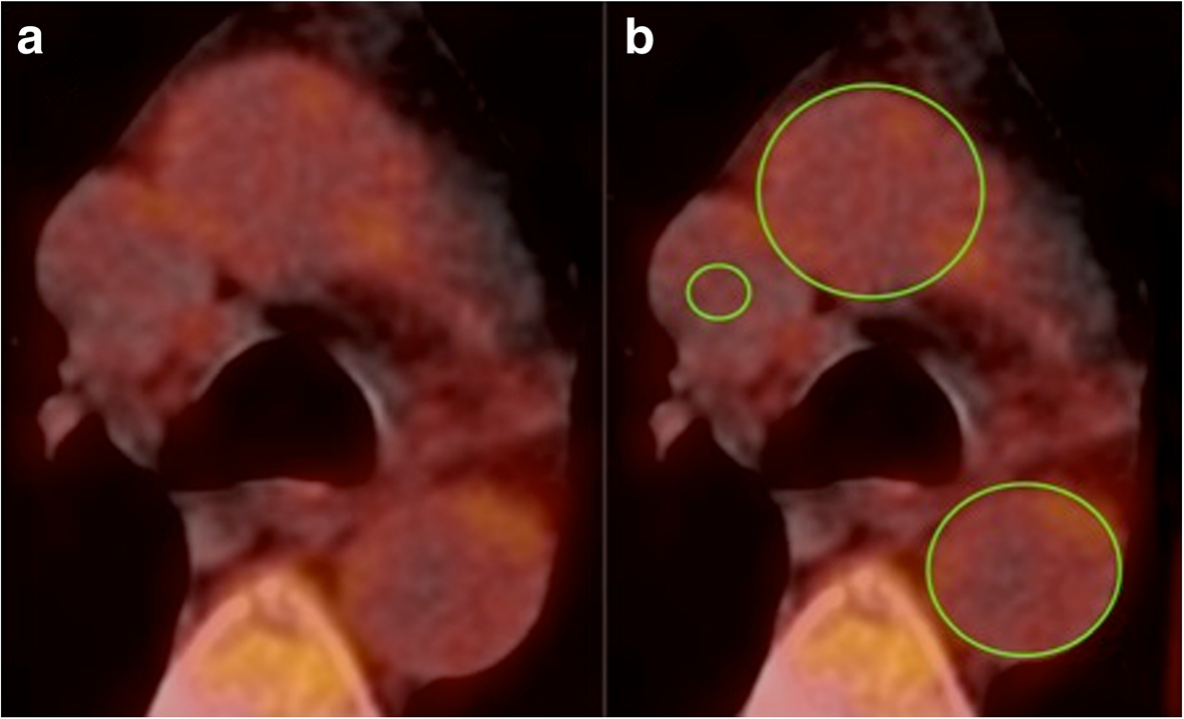
Typical fused PET/CT image. **a** Fused FDG PET/CT axial image of the aorta and superior vena cava. **b** Tissue-to-blood ratio (TBR) calculated by dividing FDG uptake in aorta (green circles) by uptake in the superior vena cava (small green circle)

^18^F-Fluorodeoxyglucose positron emission tomography co-registered with computed tomography (FDG PET/CT) imaging of the aorta and carotid arteries were performed. Full imaging methods are explained in the Additional file 1. For image analysis, Osirix open source DICOM software (v5.6, Osirix Imaging Software, Geneva, Switzerland) was used. The maximum standardized uptake value (SUVmax) of FDG within each axial PET/CT fused image of artery, containing wall and lumen, was divided by the average blood FDG concentration in the superior vena cava or jugular vein (for carotids) to yield an arterial maximum tissue-to-blood (TBR) ratio, as a quantitative measure of arterial tracer uptake, see Fig. 1. Average TBR for thoracic aorta, abdominal aorta, and entire aorta, as well as average TBR for the right and left carotid arteries combined, were calculated. Additional analysis examined the proportion of aortic slices with a TBR > 2, which was used as a threshold to define highly inflamed areas of aorta [21]. All scans were analyzed by an experienced reader, anonymized to patient identifiable information, subject group, and visit number.

Aortic calcification (defined by an attenuation threshold ≥130 Hounsfield units in 3 contiguous voxels as per the Agatston method [22]) was analyzed for each cross sectional CT scan slice of aorta [23]. The total score for the aorta was obtained by adding scores from all cross-sectional images. Emphysema severity, defined by the Perc 15 score (Hounsfield Units) which is a measure of lung density [24], was also calculated from CT scan imaging.

Data from the placebo group of the EVOLUTION trial enabled longitudinal analysis of the stability of aortic inflammation in 34 COPD patients. Aortic TBR values derived from baseline and repeat imaging at 4 months were compared.

### Hemodynamic assessments

aPWV was measured using a high fidelity micromanometer (SPC-301, Millar Instruments, Houston, Texas) and Sphygmocor device (Sphygmocor, AtCor Medical, Sydney, Australia), as previously described [25].

### Blood biomarkers of inflammation

Blood samples were analyzed for high sensitivity CRP (hsCRP), plasma fibrinogen (Klauss method), as well as total white cell count (WCC), neutrophils and eosinophils, in NHS laboratories using validated assays.

### Statistical analysis

Sample size was determined based on previous data evaluating vascular inflammation in COPD patients compared to controls [10], and this current study includes the largest to date, prospective cohort of COPD patients with vascular FDG PET imaging. Data were analyzed using SPSS software (version 23). Mixed model linear regression analyses were used to evaluate TBR data with study site as a covariate. In addition, a logistic regression model with a binary outcome response of TBR > 2 or ≤ 2, with site also as a covariate, was also performed. Mann-Whitney U test was used to compare aortic calcification to never-smokers. Blood biomarker data were log10 transformed prior to statistical analyses. Pearsons two-tailed r test or Spearman’s rho were used to evaluate correlations, and unpaired t-test and Chi-square test were used to compare demographic data to never-smokers. Data are expressed as mean ± standard deviation (SD), median (interquartile range (IQR)) where appropriate. Bar graphs (with error bars) represent mean (95% confidence intervals (CI)) values. A *p*-value < 0.05 was deemed statistically significant for all analyses.

## Results

Eighty-five COPD patients, 12 α_1_ATD-COPD patients, 12 each healthy smokers and never-smokers were studied. The demographic data for each group are summarized in Table 1. COPD patients were aged 68 ± 8 years; never-smokers 69 ± 7 years and both smokers and α_1_ATD patients were slightly younger at 62 ± 6 years and 62 ± 8 years, respectively. There were a higher proportion of women in the α_1_ATD and smokers groups, although this did not reach statistical significance. There was no difference in pack years smoked between COPD patients and smokers (43 ± 21 versus 37 ± 19 pack years, *p* = 0.36) but both groups smoked significantly more than α_1_ATD patients (19 ± 11 pack years, *p* < 0.001 for both). Sixty-eight (80%) of COPD patients and 10 (83%) of α_1_ATD patients used a combined long acting beta agonist/inhaled corticosteroid inhaler. Out of the whole study population, 4 subjects; who were COPD patients, had type 2 diabetes, which was controlled with oral hypoglycaemics. No α1ATD patients or smokers were taking statins, whereas 26 COPD patients (31%) and 2 never-smokers (17%) were. There were no differences in the lipid profile or pre-scan glucose levels between any subject groups.

**Table 1:** Demographic characteristics of subject groups

Variable		COPD *n* = 85	α_1_ATD *n* = 12	Smokers n = 12	Never smokers n = 12
Demographics					
Age (years)		68 ± 8	62 ± 8*	62 ± 6*	69 ± 7
Gender (% male)		67	73	58	83
BMI (kg/m^2^)		25.9 ± 3.9	25.0 ± 3.3	23.1 ± 2.3*	26.6 ± 2.6
Current smoker n (%)		11 (13)***	2 (17)***	12 (100)***	0
Pack years smoked		45 ± 25***	19 ± 11***	37 ± 19***	0
Statin therapy n (%)		26 (31)*	0***	0***	2 (17)
Lung function					
FEV_1_ (L)		1.37 ± 0.6***	1.47 ± 0.4***	2.84 ± 0.6	2.88 ± 0.6
FEV_1_% predicted		51 ± 20***	45 ± 16***	95 ± 17	100 ± 15
GOLD stage II/III (%)		77	83	–	–
Perc 15 score (HU)		− 890 ± 54***	− 942 ± 18***	−818 ± 23	−806 ± 36
Hemodynamic measurements					
SBP (mmHg)		137 ± 18	132 ± 11	134 ± 16	131 ± 8
DBP (mmHg)		79 ± 7	84 ± 8	84 ± 7	79 ± 7
Heart rate (bpm)		71 ± 18	76 ± 10	67 ± 16	66 ± 10
Aortic pulse wave velocity (m/s)		9.9 ± 2.6*	9.5 ± 1.8*	7.8 ± 1.8	7.9 ± 1.7
Laboratory data					
Fibrinogen (g/L)		3.4 ± 0.7*	3.1 ± 0.6	2.8 ± 0.6	2.7 ± 0.5
hsCRP (mg/L)		5.2 ± 7.0*	3.3 ± 2.3*	2.1 ± 1.4	1.2 ± 0.6
White cell count (×10^9^/L)		6.54 ± 1.83	7.01 ± 2.72	7.28 ± 2.02	5.84 ± 1.31
Neutrophils (×10^9^/L)		4.43 ± 3.6	4.68 ± 2.47	4.53 ± 1.45	3.63 ± 1.11
LDL Cholesterol (mmol/L)		2.9 ± 1.0	3.54 ± 0.4	3.1 ± 1.1	3.1 ± 1.1
Total/HDL Cholesterol (mmol/L)		3.5 ± 1.3	3.5 ± 1.3	3.5 ± 1.6	3.4 ± 1.2
Triglycerides (mmol/L)		1.4 ± 0.8	1.5 ± 1.0	1.2 ± 0.7	1.2 ± 0.5

Mean ± SD presented. Data are presented compared to never-smokers. Unpaired t-test or Chi-square test used to determine differences compared to never-smokers: ***p < 0.001, *p < 0.05 compared to never-smokers

*BMI* Body mass index, *FEV*_*1*_ Forced expiratory lung volume in 1 s, *GOLD* (Global Obstructive Lung Disease), *HU* Hounsfield Units, *SBP* systolic blood pressure, *DBP* diastolic blood pressure, *LDL* low-density lipoprotein, *HDL* high-density lipoprotein, *hsCRP* (high sensitivity C-reactive protein)

### Vascular inflammation

**Fig. 2:**
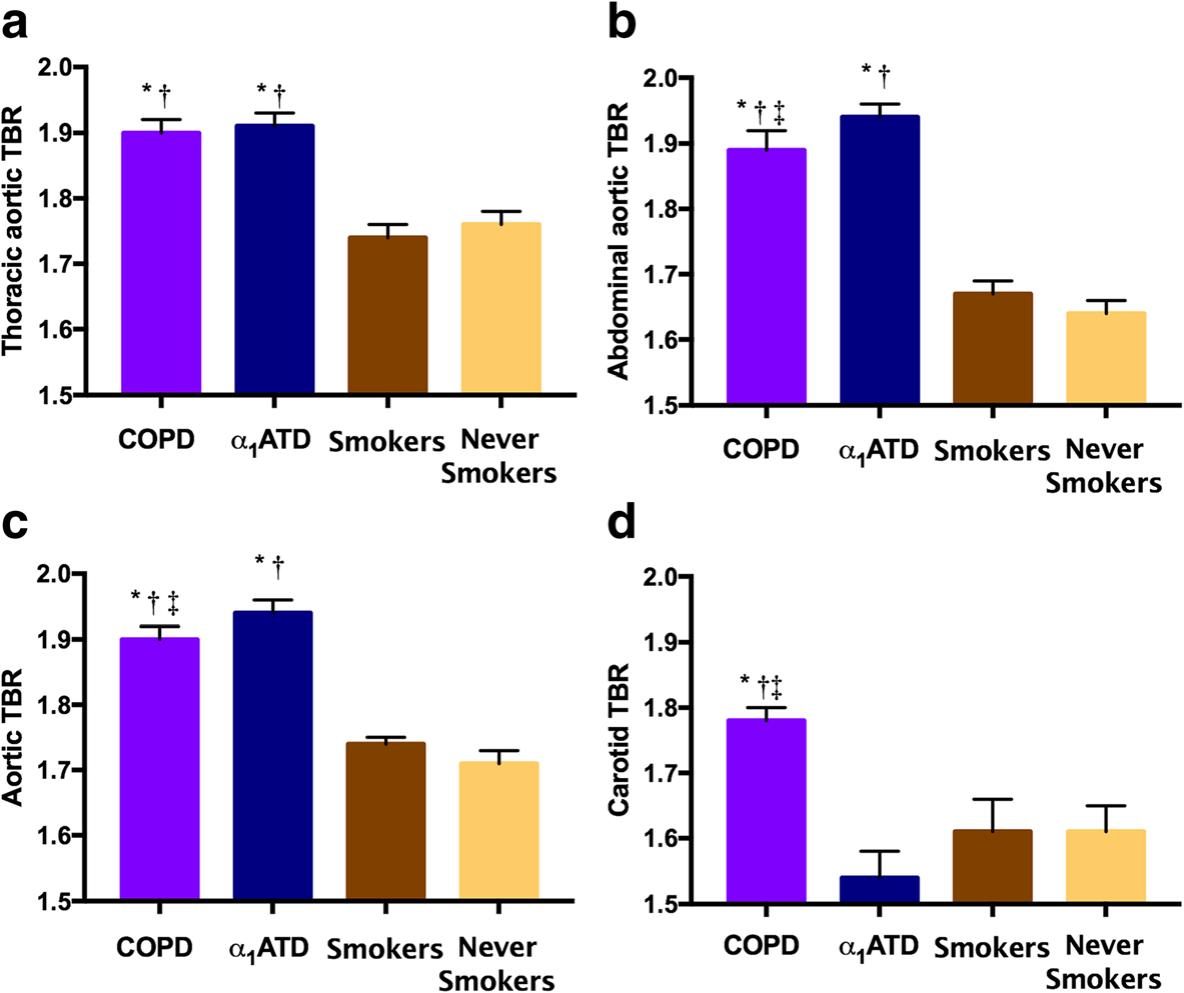
Mean tissue-to-blood ratio (TBR) in each of the aortic regions and carotid artery. **a**. Thoracic aorta, **b**. Abdominal aorta, **c**. Whole aorta, **d**. Carotid artery. Bars represent mean values and error bars represent 95% CI. Overall significance for each model was *p* < 0.001. Pairwise comparisons are illustrated by *p < 0.001 compared to never smokers, † *p* < 0.001 compared to smokers, ‡*p* < 0.01 compared to α1ATD patients

Both COPD and α_1_ATD patients had increased inflammation in the thoracic, abdominal and entire aorta in comparison to healthy smokers (*p* < 0.001 for all) and never-smokers, (p < 0.001 for all), Table 2 and Fig. 2. The greatest difference in aortic inflammation for both COPD and α_1_ATD patients, compared to never-smokers, was in the abdominal aorta, with differences in TBR of + 15%, p < 0.001, and + 18% p < 0.001, respectively. COPD and α1ATD patients also had a greater proportion of highly inflamed (TBR > 2) aortic slices (31 and 34% respectively), versus controls (smokers and never-smokers data combined), who had only 9%, p < 0.001 for both. Interestingly, α_1_ATD patients were more inflamed than COPD patients (*p* < 0.001), and there were no differences in TBR between COPD patients who were smokers and ex-smokers in any aortic region. Furthermore, aortic inflammation was evaluated at baseline and at 4-months in COPD patients who received placebo in the EVOLUTION trial, and it did not change significantly (baseline TBR: 1.89 ± 0.28 versus 4 months TBR: 1.87 ± 0.27, *p* = 0.54).

**Table 2:** Image data

Image variable	COPD n = 85	α_1_ATD n = 12	Smokers n = 12	Never smokers n = 12
Thoracic TBR	1.90 ± 0.33***	1.91 ± 0.40***	1.74 ± 0.32	1.76 ± 0.31
Abdominal TBR	1.89 ± 0.32***	1.94 ± 0.44***	1.67 ± 0.23	1.64 ± 0.24
Entire Aorta TBR	1.90 ± 0.38***	1.94 ± 0.43***	1.74 ± 0.30	1.71 ± 0.34
Carotid TBR	1.74 ± 0.22***	1.57 ± 0.46	1.61 ± 0.31	1.60 ± 0.30
Aortic calcification (Agatston Units)	2018 * (1089, 3581)	399 (12, 1468)	807 (118, 3201)	274 (0,1314)
Aortic calcification (mm^3^)	2504* (1403, 4393)	1778 (147, 5683)	926 (153, 5976)	344 (0, 2456)

Mean ± SD, median (IQR) presented. Data are presented compared to never-smokers. Mixed model linear regression analysis used to evaluate differences in TBR compared to never-smokers, Mann-Whitney U test to compare aortic calcification to never-smokers. ***p < 0.001,*p < 0.05 compared to never-smokers

*TBR* tissue-to-blood ratio

In regard of aortic calcification, an inverse association between aortic TBR and calcification was observed (rho = − 0.28, *p* = 0.04), and COPD patients had the most aortic calcification compared to never-smokers who had the least, *p* = 0.02, Table 2. Nonetheless, there were no significant differences in aortic calcification between α_1_ATD patients, smokers and never-smokers.

In the carotid artery, COPD patients had a higher TBR (1.74 ± 0.22), than all other groups, *p* < 0.001, but there were no statistically significant differences in carotid TBR between α_1_ATD patients, smokers or never-smokers.

There was no correlation between age, body mass index (BMI), or pack years smoked with aortic TBR, but carotid TBR did correlate with pack years (*r* = 0.34, *p* = 0.03). There was an association between thoracic and abdominal aortic TBR (*r* = 0.66, p < 0.001), but correlations between carotid TBR with both thoracic and abdominal TBR were non-significant (*r* = 0.11, *p* = 0.26 and *r* = 0.18, *p* = 0.05).

### Aortic stiffness

aPWV was increased in COPD and α_1_ATD patients compared to controls (both never-smokers and smokers), *p* ≤ 0.05 for all. After adjustment for mean arterial pressure and heart rate, a difference of + 1.4 m/s in aPWV (95% CI 8.83, 10.17), *p* = 0.01, between lung disease patients (COPD and α_1_ATD patients data combined) and controls (smokers and never-smokers data combined) was observed. However, aPWV was similar between the two lung disease groups, and also between the two control groups.

### Aortic inflammation, stiffness and calcification

We observed no correlation between aortic inflammation and stiffness in any subject group or the whole cohort. However, given that aortic calcification was positively correlated with aPWV (r = + 0.30, *p* = 0.001), but inversely correlated with aortic TBR, analysis of only subjects with no aortic calcification was performed. This showed a modest positive but non-significant correlation between aortic TBR and aPWV in the whole cohort (*n* = 10 subjects, *r* = + 0.37, *p* = 0.28).

### Vascular and systemic inflammation

There was a borderline significant positive correlation between abdominal aortic TBR with both WCC and neutrophils (*r* = + 0.18, *p* = 0.05 and r = + 0.17, *p* = 0.06) in the whole cohort only. In α_1_ATD patients, a correlation between fibrinogen and aortic TBR (*r* = + 0.71, *p* = 0.01) was observed, but not in COPD patients, or the whole cohort. In the carotid artery, a modest correlation with hsCRP was seen in COPD (*r* = + 0.23, *p* = 0.04), α_1_ATD patients (*r* = + 0.67, p = 0.01), and the whole cohort (*r* = + 0.41, *p* = 0.02), and in COPD patients, also a modest correlation between both neutrophils and WCC with carotid TBR (*r* = + 0.33, *p* = 0.03 and *r* = + 0.26, *p* = 0.03, respectively) was observed. In COPD patients, we also examined if there was a correlation between eosinophil counts with aortic or carotid inflammation, and no significant correlations were found.

### Vascular inflammation and COPD severity

In COPD patients, inverse correlations between abdominal aortic TBR with FEV_1_% predicted, (*r* = − 0.19, *p* = 0.07), FVC % predicted (*r* = − 0.29, *p* = 0.007) and the Perc 15 score of emphysema severity (*r* = − 0.25, p = 0.01) were observed, but there were no correlations between measures of COPD severity with thoracic aortic or carotid artery TBR.

## Discussion

The novel findings of this prospective study are that increased aortic inflammation and aortic stiffness are found in both ‘usual’ COPD *and* α1ATD-related COPD patients, compared to control smokers and never-smokers. In contrast, there was no difference in these surrogate markers of CVD between smokers and never-smokers. Moreover, COPD patients had increased carotid artery inflammation compared to all other subject groups and increased aortic calcification compared to never-smokers. We also demonstrated that aortic inflammation in COPD patients is a stable finding, over a 4-month period. Additional novel findings include the lack of a cross-sectional relationship between aortic inflammation and aortic stiffness in COPD patients. Taken together, these data suggest that cardiovascular risk in COPD patients plausibly relates to vascular inflammation and stiffening, these are distinct mechanistic entities in cross-sectional analysis at least; and these findings cannot be simply attributed to smoking.

For the first time, we have demonstrated that both COPD and α_1_ATD-related COPD patients have increased aortic inflammation compared to smokers and never-smokers without COPD. The findings of + 15% and + 18% higher aortic TBR seen in COPD and α_1_ATD patients compared to controls, is a clinically important difference, that may be a target for anti-inflammatory therapy, since intervention studies report a 6–15% reduction in TBR with anti-inflammatory treatments such as anti-tumor necrosis-α therapy, or high-dose statins (80 mg atorvastatin) [21, 26]. We also found that both COPD and α_1_ATD-related COPD patients had higher aortic TBR values than we previously reported in atherosclerosis subjects, but lower than in rheumatoid arthritis (RA), which is a condition with marked inflammation [21]. For the first time, we have also shown that COPD patients demonstrated increased carotid inflammation. These are clinically important findings since in atherosclerosis, FDG uptake is consistent with the increased metabolic activity of macrophages in inflamed plaque [6] which have a greater propensity to rupture and cause cardiovascular events than stable non-inflamed plaque [6], and retrospective studies in cancer surveillance cohorts, have demonstrated a positive, independent relationship between aortic inflammation and future cardiovascular events [7, 9]. Indeed, aortic TBR strongly predicted cardiovascular events independently of traditional risk factors including coronary calcium score (hazard ratio: 4.13; 95% CI: 1.59 to 10.76; *p* = 0.004), improved net reclassification of risk when incorporated into the Framingham risk model (27.48% (95% CI: 16.27 to 39.93) for 10% risk) and also was inversely associated with the timing of cardiovascular events (β − 0.0096; *p* < 0.0001) in analysis of 513 consecutive individuals who underwent FDG PET imaging with no prior history of CVD [7, 9]. Furthermore, carotid TBR correlated with atheromatous plaque macrophage content in endarterectomy specimens of patients with cerebrovascular disease [8], related to clinically significant culprit lesions following a recent transient ischaemic attack (TIA), and culprit carotid non-stenotic lesions identified on magnetic resolution imaging as compatible with symptomatic vessel territories [27, 28]. Carotid inflammation may therefore be an important factor in the increased risk of stroke observed in COPD patients [29] and is an area that requires further research.

Interestingly, we found no difference in vascular inflammation levels measured by FDG PET between control smokers and never-smokers, which is consistent with previous data [30]. A potential explanation for this finding is that FDG uptake and vascular calcification are inversely related [31], and more aortic calcification observed in smokers (although not statistically significant), is a possible confounding factor. A further option, is that genetic predisposition to the noxious effects of smoke in the lungs [32] *and* vasculature, may explain differences between patients with lung disease and healthy smokers, especially as α_1_ATD patients had the highest levels of aortic inflammation, despite smoking significantly less than control smokers and COPD patients. Regarding, the lack of difference in aortic stiffness between smokers and never-smokers, large hemodynamic studies such as Framingham and the Anglo Cardiff Collaborative Trial also found either no (or a modest) influence of smoking on aortic stiffness [33, 34].

Further novel findings from the current study are that regional differences in vascular bed inflammation may have different etiological factors, particularly since α_1_ATD patients had increased aortic inflammation specifically, but low levels of carotid TBR. As FDG uptake in vascular imaging can be due to atherosclerotic plaque, or arterial wall inflammation, an important hypothesis from our data is that aortic inflammation and stiffness in α1ATD may be due to non-atherosclerotic vascular changes, since carotid artery inflammation (which in a previous study histologically corroborated with atheromatous plaque activity [8]) was low in these patients and in contrast to usual COPD patients, α_1_ATD-COPD patients did not have significant aortic calcification. Interestingly, α_1_ATD patients demonstrated the highest levels of inflammation in the abdominal aorta, and a relationship between measures of COPD severity and abdominal aortic TBR was observed. The mechanical effects of hyperinflation [35] which is associated with haemodynamic effects [36], intra-thoracic pressure swings associated with obstructive lung disease [37], and coughing and snoring [38] are all potential explanations. It is also possible that we have overlooked an unknown shared pathological mechanism, between aortic wall disorders and COPD and α_1_ATD-related COPD. Mediators of inflammatory extracellular matrix degradation, elastin loss and chronic inflammation are recognized in the pathogenesis of emphysema and COPD [39], as well as aortic dilatation, stiffening and aneurysm formation. Indeed, we found that both COPD and α_1_ATD patients compared to controls had increased aortic stiffness which is consistent with previous data [11, 40]. This is clinically relevant given the independent association between aPWV and cardiovascular events and mortality [12].

Increased aortic inflammation and stiffness observed in α_1_ATD patients with COPD, suggests that COPD per se may be associated with these adverse aortic changes and that these patients are also at increased cardiovascular risk. This is interesting since previous data regarding cardiovascular risk in α_1_ATD, unlike in usual COPD studies, are inconsistent. In the Copenhagen City Heart Study, α_1_ATD was associated with reduced blood pressure in ischaemic heart disease patients, but only 6 subjects had the PiZ phenotype (which was a tiny fraction of the population cohort, < 0.05%), and their lung function was not reported [41]. In contrast, it is reported that low levels of the α_1_AT protein are associated with atherosclerosis progression, defined by change in coronary artery luminal diameter, measured by quantitative angiography, in subjects with established vascular disease, who had undergone previous coronary artery bypass surgery [42].

A further new finding of the current study is that we observed no significant relationship between aortic inflammation and stiffness in any subject group. In contrast, we previously observed a correlation between the reduction in aortic inflammation and reduction in aortic stiffness following anti-TNF therapy in rheumatoid arthritis patients [21, 43]. Our data suggests that aortic inflammation and stiffness are either two distinct mechanisms of increased vascular risk in COPD, or there is a temporal link between them, which is not seen in this cross-sectional analysis. Aortic inflammation does precede calcium deposition in the same anatomical location of the aortic wall [44], and aortic calcification is associated with aortic stiffness in systolic hypertension subjects and in COPD patients, which is consistent with our data [45, 46]. Interestingly, we found that usual COPD patients had significantly more aortic calcification compared to age matched never-smokers, further supporting the notion of premature vascular ageing, and increased cardiovascular risk associated with COPD [47]. Furthermore, experimental data in pre-clinical mouse models, showed that pro-inflammatory pathways regulate elastinolysis and can trigger vascular osteogenesis [48]. Given that tracer uptake is diminished in regions of calcification [31], the finding that COPD patients had increased aortic FDG uptake, *and* aortic calcification compared to controls does suggest that aortic inflammation levels would be even higher in COPD patients, before calcification was established. We speculate therefore, that aortic elastin degradation due to inflammatory processes may be the process linking aortic inflammation, stiffening, and calcification in COPD.

The mechanistic importance of systemic inflammation in vascular inflammation in COPD is uncertain. Our finding of a correlation between hsCRP and carotid TBR in all subject groups is consistent with published data [49]. However, in cross-sectional analysis at least, it seems that systemic inflammatory markers are poor surrogate markers of aortic inflammation in COPD, and of note, other studies have shown a lack of association between systemic inflammation and aortic stiffness in COPD [16].

The strengths of this study are that we have used a reliable, state of the art imaging technique to examine in-vivo anatomically-localized vascular inflammation [6, 50], and enabled assessment of its relationship with vascular stiffness and systemic inflammation in the largest prospective cohort of COPD patients with PET vascular imaging. The main limitation is its cross-sectional nature, which does not permit any assessment of causality. A further potential limitation is that we selected α_1_ATD patients as a positive, COPD lung disease control group, and although these patients had significantly lower smoking history, it was hard to find α_1_ATD patients with COPD who were never-smokers. Similarly, it was difficult to exactly match the four subject groups for age and gender, and although there were no statistically significant differences across the groups, α_1_ATD patients and smokers tended to be younger and have a higher proportion of women than the usual COPD and never-smoker groups. Furthermore, the influence on our results of respiratory medication use such as inhaled corticosteroids, as well as the fact a sizeable proportion of COPD patients were already on statins needs consideration. However, one would expect these medications to reduce vascular inflammation and stiffness, therefore diminishing the observed difference between COPD patients and controls [51]. Another point of consideration is that this was a mechanistic observational study involving ionizing radiation. Therefore we used the smallest number of controls possible to test our hypothesis. However, our sample size is in keeping with other FDG PET studies of this nature [10, 28]. Finally, we used normalized FDG uptake, which is a measure of cellular glycolytic activity, as a surrogate measure of arterial wall inflammation [6]. However, FDG is a non-specific tracer, taken up by all metabolically active cells, and therefore does not dissect the different cellular profiles involved in vascular inflammation in usual COPD and α_1_ATD patients, whether there are differences between these groups, or compared to atherosclerosis subjects. More targeted tracers, for example in atherosclerosis imaging, 11C-PK11195 and 68Ga- DOTATATE have been used to evaluate macrophage activity [7], may be of value for evaluating specific interventions in the future.

## Conclusions

COPD patients have increased aortic inflammation and stiffness, irrespective of tobacco consumption, which are plausible mechanisms mediating increased cardiovascular risk, associated with COPD. COPD patients also demonstrated increased carotid inflammation and aortic calcification, which further demonstrates the adverse vascular characteristics of these patients, and the need for action to improve understanding of cardiovascular risk in COPD to positively influence outcomes. Moreover, our data raises interesting questions regarding the etiology of vascular inflammation affecting different arterial territories in COPD, which is a topic that should be addressed in further studies. Additionally, trials with these non-invasive vascular markers, and therapeutic intervention to attenuate them, with the aim to reduce cardiovascular risk, are needed.

## Additional file

Additional file 1: Scan Image Protocols. (DOCX 92 kb)

## Abbreviations

aPWV: Aortic (carotid-femoral) pulse wave velocity
COPD: Chronic obstructive pulmonary disease
TBR: Tissue to blood ratio
α_1_ATD: Alpha-1 antitrypsin deficiency
