# Supplementary material for: Vascular inflammation and aortic stiffness: potential mechanisms of increased vascular risk in chronic obstructive pulmonary disease
Source: Respir Res. 2018 May 24;19:100. doi: 10.1186/s12931-018-0792-1 (PMC5968523; doi:10.1186/s12931-018-0792-1)
Supplement: Supplementary file 1 — Scan Image Protocols. (DOCX 92 kb) [file 12931_2018_792_MOESM1_ESM.docx]

**Supplementary material for**

**‘Vascular Inflammation and Aortic Stiffness: Potential Mechanisms of Increased Vascular Risk in Chronic Obstructive Pulmonary Disease’**

**Short title and name:** **Mechanisms of vascular risk in COPD (Fisk)**

Marie Fisk PhD^1^, Joseph Cheriyan FRCP^1,2^, Divya Mohan PhD^3,6^, Carmel M McEniery^1^, Julia Forman^2^, John R Cockcroft^4^, James HF Rudd^5^, Ruth Tal-Singer^6^,

Nicholas S Hopkinson^3^, Michael I Polkey^3^, Ian B Wilkinson^1,2^

1. Division of Experimental Medicine and Immunotherapeutics, University of Cambridge, UK
2. Cambridge Clinical Trials Unit, Cambridge University Hospitals NHS Foundation Trust, Cambridge, UK
3. NIHR Respiratory Biomedical Research Unit, Royal Brompton & Harefield NHS Foundation Trust and Imperial College, London, UK
4. Department of Cardiology, Wales Heart Research Institute, Cardiff University, Cardiff, UK
5. Division of Cardiovascular Medicine, University of Cambridge & Cambridge University Hospitals NHS Foundation Trust, Cambridge, UK
6. GSK R&D, King of Prussia, Pennsylvania, USA

**Corresponding Author:**

Dr Marie Fisk, Box 98, Level 3, ACCI Building, Cambridge University Hospitals NHS Foundation Trust, Hills Road, CB2 0QQ.

Tel: +44 (0) 1223-336-806. Fax: +44 (0) 1223-296-006

**Scan Image Protocols**

Scans were performed at the PET/CT unit, Addenbrookes Hospital and Imanova Centre, Hammersmith. A General Electric Lightspeed VCT (Milwaukee, Wisconsin) scanner was used in Addenbrookes and a Siemens Biograph (Munich, Germany) scanner in Imanova. Reconstruction algorithms were used to minimise variability and provide clinical quality, comparable image data for analysis.

Subjects were required to fast for 6 hours prior to the scan and to avoid any strenuous exercise in the preceding 24 hours to limit muscle uptake of tracer. Any metal objects were removed prior to scanning and only clothing without metallic fastenings allowed during imaging. Blood glucose levels had to be less than 11mmol/L to proceed with the scan.

A dose of approximately 240Mbq ^18^F-Fluorodeoxyglucose (FDG) was injected, followed by 10mls flush of normal saline. At 90 minutes post injection, the non-contrast CT scan of the aorta was performed from arch to bifurcation, followed by a PET acquisition (3x10 minute bed positions in 3D mode).

The carotid artery scan was then undertaken at approximately two hours after FDG injection. The head and neck were placed in a holder and a non-contrast CT scan of the neck undertaken. This was immediately followed by a single bed PET scan acquired in 3D mode for 15 minutes, where the external auditory meatus was the upper anatomical landmark of the scan. The CT scans were used for attenuation correction and anatomical co-registration. PET data for the aorta and carotids was reconstructed using the default 3D iterative algorithm on each scanner. All scans were reported by consultant radiologists and any unusual finding assessed by the study team.
